# A Method for Amplicon Deep Sequencing of Drug Resistance Genes in Plasmodium falciparum Clinical Isolates from India

**DOI:** 10.1128/JCM.00235-16

**Published:** 2016-05-23

**Authors:** Pavitra N. Rao, Swapna Uplekar, Sriti Kayal, Prashant K. Mallick, Nabamita Bandyopadhyay, Sonal Kale, Om P. Singh, Akshaya Mohanty, Sanjib Mohanty, Samuel C. Wassmer, Jane M. Carlton

**Affiliations:** a Center for Genomics and Systems Biology, Department of Biology, New York University, New York, New York, USA; b National Institute of Technology, Raurkela, Odisha, India; c National Institute of Malaria Research, Indian Council of Medical Research, Dwarka, New Delhi, India; d Institute of Life Sciences, Bhubaneswar, Odisha, India; e Ispat General Hospital, Raurkela, Odisha, India; f Department of Microbiology, Division of Parasitology, New York University School of Medicine, New York, New York, USA; University of Texas Medical Branch

## Abstract

A major challenge to global malaria control and elimination is early detection and containment of emerging drug resistance. Next-generation sequencing (NGS) methods provide the resolution, scalability, and sensitivity required for high-throughput surveillance of molecular markers of drug resistance. We have developed an amplicon sequencing method on the Ion Torrent PGM platform for targeted resequencing of a panel of six Plasmodium falciparum genes implicated in resistance to first-line antimalarial therapy, including artemisinin combination therapy, chloroquine, and sulfadoxine-pyrimethamine. The protocol was optimized using 12 geographically diverse P. falciparum reference strains and successfully applied to multiplexed sequencing of 16 clinical isolates from India. The sequencing results from the reference strains showed 100% concordance with previously reported drug resistance-associated mutations. Single-nucleotide polymorphisms (SNPs) in clinical isolates revealed a number of known resistance-associated mutations and other nonsynonymous mutations that have not been implicated in drug resistance. SNP positions containing multiple allelic variants were used to identify three clinical samples containing mixed genotypes indicative of multiclonal infections. The amplicon sequencing protocol has been designed for the benchtop Ion Torrent PGM platform and can be operated with minimal bioinformatics infrastructure, making it ideal for use in countries that are endemic for the disease to facilitate routine large-scale surveillance of the emergence of drug resistance and to ensure continued success of the malaria treatment policy.

## INTRODUCTION

Mortality due to malaria has decreased by half since 2000, and the global incidence has decreased by 37%, yet the disease remains a significant public health burden (1). Countries with large populations in particular struggle to combat the disease with underresourced and inadequate health care facilities. India, with its population of 1.2 billion, contributes a significant number of malaria cases each year, although it has been making significant progress toward the goal of zero malaria cases set by the National Vector Borne Disease Control Programme. Indeed, India is now in the control phase of a four-phase path toward a malaria-free status, with an aim of reaching preelimination status by 2017 (2).

One of the major threats to global malaria control is the rise and spread of parasite resistance to antimalarial drugs. In order to achieve preelimination status by 2017, India needs to scale up interventions to ensure the efficacy of first-line antimalarial therapy and monitor the emergence of drug-resistant parasites, particularly in areas with the highest disease burden. Effective monitoring of drug efficacy and surveillance of drug resistance in regions endemic for malaria require an integrated approach comprising *in vivo* drug trials, *in vitro*/*ex vivo* drug efficacy assays, and tracking of molecular markers of drug resistance. *In vivo* testing is the gold standard to measure clinical efficacy of antimalarial drugs, but it is expensive and difficult to implement on a regular basis and on a large scale. *In vitro* assays provide an understanding of intrinsic resistance; however, they are labor-intensive and require laboratory infrastructure for *in vitro* culture. In contrast, molecular markers of resistance offer a simpler alternative for routine surveillance. Although these do not take into account host immunity and drug pharmacokinetics, molecular markers can help track the emergence of drug-resistant malaria parasites before they become clinically evident (3).

A number of molecular genotyping techniques have been developed and implemented over the last few decades for monitoring drug resistance in clinical isolates. Traditional methods include restriction fragment length polymorphism (RFLP) analysis (4), molecular beacons (5), real-time PCR (6, 7), dot blot probe hybridization (8), and single-nucleotide primer extension (9). More recent high-throughput methods include high-resolution DNA melting (HRM) (10, 11), a single-nucleotide polymorphism (SNP)-based custom genotyping assay (12, 13), a TaqMan allelic discrimination assay (14), mass spectrometry-based SNP genotyping (15), a ligase detection reaction fluorescent microsphere (LDR-FM) assay (16), and loop-mediated isothermal amplification (LAMP) (17). Each method comes with its own advantages and disadvantages, so researchers can choose a suitable technique based on the requirements, facilities, and resources available. A common limitation of these techniques is that while they allow the detection of known resistance alleles of a particular gene, they do not facilitate the discovery of novel genetic polymorphisms that could be involved in drug resistance. Sanger sequencing of DNA polymorphisms provides one method for identifying novel drug resistance polymorphisms and has served as a gold standard for the validation of molecular genotyping. However, its application to large-scale surveillance is limited by low throughput, inability to detect polymorphisms at minor frequencies, and the high cost of reagents.

As a result of its high throughput, resolution, and scalability, next-generation sequencing (NGS) has been harnessed to develop genomic tools for understanding the genetic diversity and evolution of pathogens, characterizing transmission networks, and importantly, detecting drug resistance. In addition, the development of modestly priced benchtop sequencing instruments with high turnover rates makes it possible to integrate NGS into countries that are endemic for the disease. There are a number of publications that describe the application, implementation, and running costs for the most commonly used benchtop instruments, including performance comparisons based on technical specifications, data quality, throughput, and overall performance, and these can be referenced for further information (18–23). While whole-genome sequencing (WGS) of pathogen field samples is ideal, it is impractical for analyzing hundreds of clinical samples given the current high costs of sequencing reagents, and data storage and manipulation requirements. In contrast, amplicon sequencing involves targeted amplification of one or several loci, followed by NGS to generate a large number of sequencing reads that can be used to assess the genetic diversity in clinical samples. This method is more suitable for analysis of clinical samples, as it requires much less DNA than that with WGS and can utilize DNA obtained from filter paper blood spots or small-volume collections without the need for preprocessing of samples to remove human DNA contamination (24). Amplicon sequencing of single genomic loci has been used to identify parasite haplotypes in Plasmodium falciparum by 454 pyrosequencing of the circumsporozoite protein gene (25, 26), and to detect genetic signatures of Plasmodium vivax relapse by Ion Torrent Personal Genome Machine (PGM) sequencing of the merozoite surface protein-1 locus (27).

In this paper, we describe a novel protocol for Ion Torrent PGM amplicon sequencing of a panel of P. falciparum drug resistance genes, based upon a method developed by Daum et al. (28, 29) to evaluate Mycobacterium tuberculosis drug resistance in clinical strains. Our panel of P. falciparum drug resistance markers includes genes implicated in resistance to artemisinin derivatives, sulfadoxine-pyrimethamine, chloroquine, and other drugs. The protocol was tested first on 12 publicly available P. falciparum reference strains and subsequently applied to clinical isolates from patients in India, with the ultimate goal of establishing it as a routine surveillance method in a country that is endemic for the disease.

## MATERIALS AND METHODS

### Ethics statement.

The clinical samples examined in this study were collected after ethical approval from the New York University institutional review board (study no. i10-00173) and the ethics committee of Ispat General Hospital, Raurkela, India.

### P. falciparum reference strains.

Genomic DNA from P. falciparum strains NF54 E (MRA-1000G), TM91C235 (MRA-206G), Dd2 (MRA-150G), 7G8 (MRA-152G), HB3 (MRA-155G), W2 (MRA-157G), K1 (MRA-159G), V1/S (MRA-176G), D10 (MRA-201G), GB4 (MRA-925G), D6 (MRA-285G), and FCB (MRA-309G) was obtained from the Malaria Research and Reference Reagents Resource (MR4, Manassas, VA, USA). The geographical location and drug resistance phenotype of the strains (where known) are shown in Table 1.

**TABLE 1:** Geographical origin, source, and drug resistance phenotype of 12 P. falciparum laboratory reference strains and 16 P. falciparum clinical isolates from Raurkela, India

Sample name(s)	Geographical origin	Source	Drug resistance phenotype^*a*^			Reference(s)
			CQ	SUL	PYR	
NF54 E	The Netherlands; presumed African origin	MR4	S	S	S	85
TM91C235	Thailand	MR4	R	R	R	86
Dd2	Indochina	MR4	R	R	R	85
7G8	Brazil	MR4	R	S	R	85
HB3	Honduras	MR4	S	S	R	85
W2	Indochina	MR4	R	R	R	86
K1	Thailand	MR4	R	R	R	85
V1/S	Vietnam	MR4	R	R	R	85
D10	Papua New Guinea	MR4	S	S	S	55, 72, 87
GB4	Ghana	MR4	R	NA	NA	88
D6	Sierra Leone	MR4	S	S	S	85
FCB	Colombia	MR4	R	NA	S	85
RKL9610, RKL10551, RKL10868, RKL12330, RKL50072, RKL50617, RKL58812, RKL58925, RKL59175, RKL59290, RKL59796, RKL60303, RKL60308, RKL60355, RKL61495, RKL64249	Ispat General Hospital, Raurkela, India	Clinical isolates	NA	NA	NA	

a S, sensitive; R, resistant; NA, not available.

### P. falciparum clinical isolates.

Parasite isolates were obtained from 16 patients admitted to Ispat General Hospital (IGH) in Raurkela, Odisha, India, between August 2014 and January 2015, and as part of the National Institutes of Health-funded International Center of Excellence in Malaria Research (ICEMR) (30). Malaria transmission in the Raurkela area is seasonal meso-/hyperendemic, with P. falciparum as the major infecting species. Patients were enrolled after obtaining informed consent and treated with antimalarials, as per the Indian national guidelines (31). An average of 5 ml of blood was collected in EDTA Vacutainers (Thermo Fisher Scientific, MA, USA) from each subject and centrifuged at 1,500 × *g* for 15 min at room temperature. Plasma and platelets were removed and pelleted infected red blood cells stored at −80°C until DNA extraction. Four of the samples were leukocyte depleted before storage: one sample (RKL59796) was passed through a CF11 filtration column (Sigma-Aldrich, MO, USA), and three samples (RKL64249, RKL50072, and RKL12330) were passed through Plasmodipur filters (EuroProxima, The Netherlands). DNA was extracted from each of the 16 samples using the QIAamp DNA blood MIDI kit (Qiagen, CA, USA), according to the manufacturer's instructions.

### Primer design.

Oligonucleotide primers (see Table S1 in the supplemental material) spanning six P. falciparum genes, kelch protein *Pfk13* (PF3D7_1343700), chloroquine resistance transporter *Pfcrt* (PF3D7_0709000), bifunctional dihydrofolate reductase-thymidylate synthase *Pfdhfr-ts* (PF3D7_0417200), hydroxymethyldihydropterin pyrophosphokinase-dihydropteroate synthetase *Pfpppk-dhps* (PF3D7_0810800), multidrug resistance protein *Pfmdr1* (PF3D7_0523000), and multidrug resistance-associated protein-1 *Pfmrp1* (PF3D7_0112200) were designed with Primer3 (32) using the 3D7 reference assembly version 3.0 from the malaria database PlasmoDB (33). The Integrated DNA Technologies (IDT) OligoAnalyzer software was used to calculate the melting temperature (*T_m_*), and for homo- and heterodimer analysis, and NCBI Primer-BLAST (34) was used to test species specificity. All oligonucleotides were synthesized by Sigma-Aldrich, USA.

### Gene amplification.

Amplification reactions were carried out with 5 ng of MR4 reference strain DNA or 2 μl of clinical isolate DNA as the template in a 25-μl reaction volume with Phusion high-fidelity PCR master mix, HF buffer (Thermo Fisher Scientific), and a 0.2 μM concentration of forward and reverse primers. The cycling parameters used to amplify all loci were as follows: 98°C for 30 s, 35 cycles of (98°C for 10 s and 64°C for 5 min 30 s) (35), with a final extension at 64°C for 10 min. Amplicons were visualized on a 1% agarose gel stained with ethidium bromide, using a GeneRuler 1-kb Plus DNA ladder (Thermo Fisher Scientific). PCR products from a single sample were pooled and purified using the QIAquick PCR purification kit (Qiagen, CA, USA) and quantified using a Qubit double-stranded DNA (dsDNA) HS assay kit (Thermo Fisher Scientific) on the Qubit 2.0 fluorometer (Thermo Fisher Scientific).

### Library preparation and amplicon sequencing.

A total of 500 ng of purified pooled PCR product from each sample was used to prepare fragment libraries using the NEBNext Fast DNA fragmentation and library prep set for Ion Torrent (New England BioLabs, MA, USA). Ion Xpress barcode adapters 1 to 28 (Thermo Fisher Scientific) were used to facilitate multiplexing of 28 samples for each sequencing run. Libraries were size selected using Agencourt AMPure XP beads (Beckman Coulter, CA, USA) and amplified with Q5 polymerase (New England BioLabs). Qualitative assessment of the libraries was carried out on the Agilent 2200 TapeStation system (Agilent Technologies, CA, USA) using high-sensitivity D1000 reagents (Agilent Technologies), to ensure proper size selection and absence of adaptor/primer dimers. Fragment libraries were then quantified on a Roche LightCycler 480 instrument II (Roche Diagnostics, Indianapolis, IN, USA) using the complete library quantification kit optimized for the Roche LightCycler 480 (Kapa Biosystems, MA, USA), and an equimolar pool of barcoded libraries was created. The library pool was templated on Ion Sphere particles using the Ion PGM Hi-Q OT2 kit on the Ion OneTouch 2 system (Thermo Fisher Scientific). Template-positive Ion Sphere particles were then enriched using the Ion OneTouch ES and loaded onto an Ion 318 Chip using the Ion Torrent PGM Hi-Q sequencing kit for 200-bp sequencing (Thermo Fisher Scientific).

### Data processing.

Sequencing data were analyzed using the Ion Torrent platform software (Torrent Suite version 4.6). Raw reads were demultiplexed and filtered using standard quality filtering parameters by the Torrent Suite pipeline software. Read quality was assessed using the Torrent Suite FastQC plugin version 0.10.1, and high-quality reads were aligned to the reference using the Torrent Mapping Alignment Program version 4.0. The reference data file used was a multi-FASTA file containing the complete gene sequence and 300-bp flanking regions for each amplicon target gene from the P. falciparum 3D7 genome using the PlasmoDB database (33). Sequencing depth and coverage across all gene targets were obtained using the Coverage Analysis plugin (version 4.6.0.3) (see Table S2 in the supplemental material).

### Variant calling and annotation.

Variant calling (see Table S3 in the supplemental material) was performed using low-stringency parameters using the Ion Torrent Variant Caller plugin (version 4.6.0.7). SNP calls were characterized using the P. falciparum 3D7 annotation and subsequently processed using custom filtering criteria (see Table S4 in the supplemental material). Given the high average read depth (700×), variants with a read coverage of <100, quality score of <100, and SNP coverage of ≤ reads were discarded. SNP annotation was performed using in-house perl and MS Excel VBA scripts (available upon request). In the case of multiple-nucleotide polymorphisms (MNPs) affecting the same codon, if the difference in the SNP allele frequencies was <40%, they were merged to calculate the resulting codon change; if the difference was >40% and the allele frequency of one of the MNPs was <10%, only the SNP with highest allele frequency was used to determine the codon change. Only high-quality variants were used for haplotype generation. At positions where no variants were reported, we confirmed the presence of the reference allele by obtaining raw nucleotide counts from corresponding BAM files using BEDTools (36). Variant calls with an SNP allele frequency of <90% were marked as heterozygous and subsequently used to classify samples as mixed infections containing more than one parasite genotype.

## RESULTS

### Design of a protocol for amplicon sequencing of P. falciparum drug resistance genes.

We developed a high-throughput protocol for multiplexed amplicon sequencing of a panel of six P. falciparum genes from clinical isolates. We chose genes associated with different types of antimalarial drug resistance in P. falciparum, as follows: (i) mutations in the propeller domain of the kelch gene *Pfk13* are associated with slow clearance of parasites in patients treated with ACT and confer resistance to artemisinin *in vitro*; (ii) mutations in the folate biosynthesis pathway enzymes dihydrofolate reductase (*Pfdhfr*) and dihydropteroate synthetase (*Pfdhps*) are associated with resistance to the antifolates sulfadoxine and pyrimethamine; (iii) mutations in transporters in the digestive vacuole of malaria parasites, including the chloroquine resistance transporter (*Pfcrt*) and multidrug resistance protein-1 (*Pfmdr1*) are major determinants of chloroquine resistance; and (iv) mutations in the parasite plasma membrane ABC transporter (*Pfmrp1*) are also associated with resistance to multiple antimalarial drugs in P. falciparum. Single primer pairs were designed to amplify almost the entire coding regions (2 to 3 kb) of *Pfk13*, *Pfcrt*, *Pfdhfr*, and *Pfdhps*, whereas *Pfmdr1* and *Pfmrp1* each required two sets of primers (see Table S1 in the supplemental material). We developed a two-step PCR protocol (see Materials and Methods) to amplify all loci simultaneously from a single patient sample, as well as an amplicon sequencing protocol optimized using DNA from several P. falciparum laboratory reference strains (Table 1). The reference strains were chosen because they have been studied extensively and exhibit well-characterized polymorphisms and drug resistance phenotypes, and they are available from the Malaria Reference and Reagent Resource Center (MR4). The protocol was designed such that amplicons from a single sample could be pooled and purified in a single tube, fragmented, and barcoded to allow multiplexing of six genes from several samples in a single NGS run. Upon testing and validating the protocol using 12 P. falciparum reference strains, we established its use for genotyping of field isolates by amplicon sequencing of 16 P. falciparum clinical samples obtained from patients at Ispat General Hospital in Raurkela, in the state of Odisha, India (Table 1).

### Sequencing data overview and validation.

**FIG 1:**
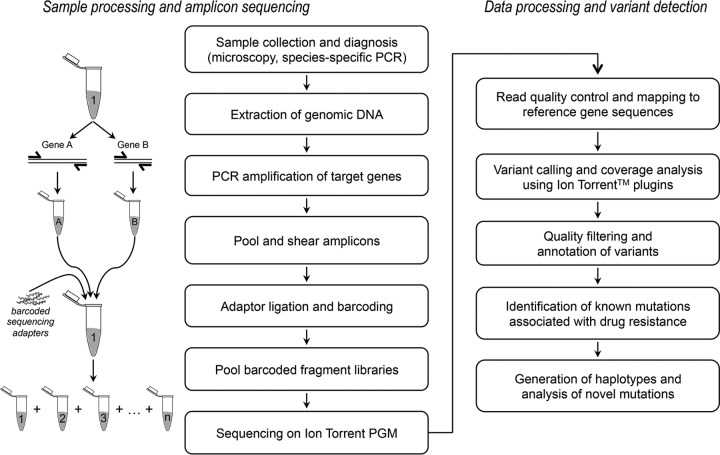
Experimental workflow used in this study, from sample collection and processing, library preparation, amplicon sequencing, and subsequent data analysis.

**FIG 2:**
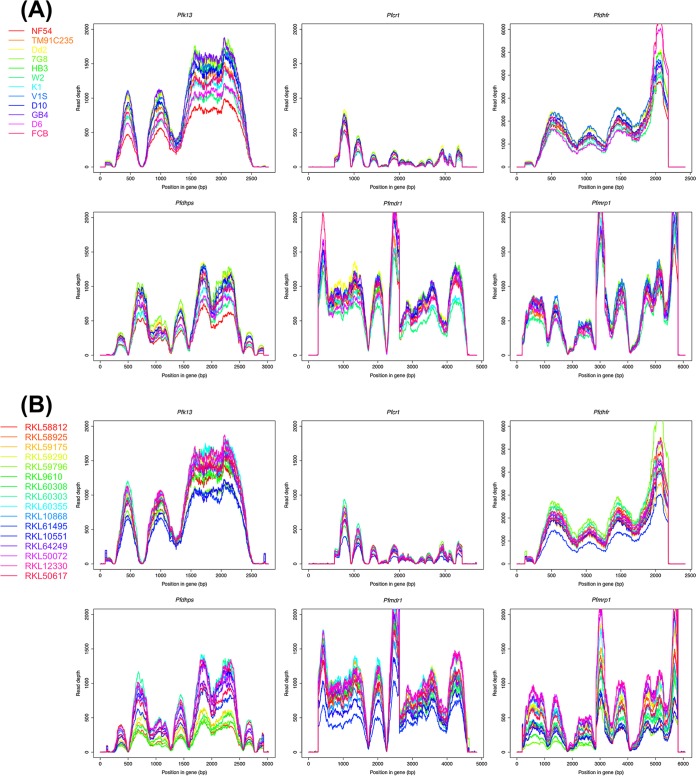
Sequencing coverage of drug resistance genes in P. falciparum reference strains and clinical isolates. Plots show sequencing read depth (*x* axis) across the amplicon length (*y* axis) for each of the six drug resistance genes. (A) A total of 12 P. falciparum reference strains from MR4 are shown, with each line corresponding to a particular reference strain shown in the key on the left. (B) A total of 16 P. falciparum clinical isolates from Raurkela, India, are shown, with each line corresponding to a particular clinical isolate shown in the key on the left.

We designed the protocol for multiplexed sequencing of amplicons on an Ion Torrent PGM (Fig. 1) using 200-bp read chemistry and the 318 Chip. We obtained >2.5 million usable reads from a single run, with roughly 92,000 reads per sample and a mean read length of 168 bp (see Table S2 in the supplemental material). All data were aligned against the P. falciparum 3D7 strain, with an average alignment rate of 95.5% and a mean depth of coverage of >700× for each sample (the cumulative length of the target genes was 20 kb). Coverage plots depicting read depth across the length of each target gene revealed comparable read distribution between P. falciparum reference strains (Fig. 2A) and clinical isolates (Fig. 2B), indicating that there was no sequencing bias associated with the clinical samples. The depth of coverage was comparable across all target genes, except the *Pfcrt* gene, most likely due to the presence of 12 high-AT homopolymer-rich introns in this gene, which are prone to sequencing errors. Variant calling and filtration generated a total of 342 high-quality coding SNPs (see Table S4 in the supplemental material) and 209 noncoding SNPs in the 28 P. falciparum samples. In order to validate that our method was able to reliably detect known variations, we compared the data with previously characterized mutations for the 12 P. falciparum reference strains in the data set. In the absence of reported variants at selected positions, we confirmed the presence of the reference allele by analyzing the raw data. Our results showed 100% concordance with previously published data for all laboratory reference strains. Along with the mutations identified at key positions associated with drug resistance, the genes exhibited additional substitutions. The 39 additional SNPs (32 nonsynonymous and 7 synonymous) were distributed across the six amplicons as follows: two in *Pfk13*, two in *Pfdhps*, three in *Pfdhfr*, nine in *Pfmrp1*, 11 in *Pfcrt*, and 12 in *Pfmdr1* (see Table S5 in the supplemental material). SNP haplotypes were deduced from key resistance-associated alleles in the six target genes for the 12 reference strains and 16 clinical samples from India (Table 2). The implications of the important resistance mutations and haplotypes identified in our data set are described in the context of each drug with which they are associated.

**TABLE 2:** Summary of SNPs implicated in drug resistance from six P. falciparum genes, *Pfcrt*, *Pfdhfr*, *Pfdhps*, *Pfmdr1*, *Pfmrp1*, and *Pfk13* in 12 reference strains and 16 clinical isolates from India^*a*^

Strain or isolate	Amino acid(s) at key position in indicated gene (with amino acid for P. falciparum 3D7 indicated below codon no.)																												
	*Pfcrt*					*Pfdhfr*					*Pfdhps*					*Pfmdr1*					*Pfmrp1*					*Pfk13*			
	72 C	73 V	74 M	75 N	76 K	16 A	51 N	59 C	108 S	164 I	436 S	437 G	540 K	581 A	613 A	86 N	184 Y	1034 S	1042 N	1246 D	191 H	437 S	876 I	1390 F	1466 K	493 Y	539 R	543 I	580 C
Reference strain																													
NF54 E	C	V	M	N	K	A	N	C	S	I	S	G	K	A	A	N	Y	S	N	D	H	S	I	F	K	Y	R	I	C
D6	C	V	M	N	K	A	N	C	S	I	**A**	**A**	K	A	A	N	Y	S	N	D	H	S	I	F	K	Y	R	I	C
D10	C	V	M	N	K	A	N	C	S	I	S	**A**	K	A	A	N	Y	S	N	D	H	S	I	F	K	Y	R	I	C
HB3	C	V	M	N	K	A	N	C	**N**	I	S	**A**	K	A	A	N	**F**	S	**D**	D	H	S	I	F	K	Y	R	I	C
Dd2	C	V	**I**	**E**	**T**	A	**I**	**R**	**N**	I	**F**	G	K	A	**S**	**F/Y**	Y	S	N	D	**Y**	**A**	**V**	**I**	K	Y	R	I	C
FCB	C	V	**I**	**E**	**T**	**V**	N	C	**T**	I	S	**A**	K	A	A	**Y**	Y	S	N	D	**Y**	**A**	**V**	**I**	K	Y	R	I	C
GB4	C	V	**I**	**E**	**T**	A	N	C	S	I	S	G	K	A	A	**Y**	**F**	S	N	D	H	S	I	F	K	Y	R	I	C
K1	C	V	**I**	**E**	**T**	A	N	**R**	**N**	I	S	G	K	**G**	A	**Y**	Y	S	N	D	**Y**	**A**	**V**	F	K	Y	R	I	C
TM91C235	C	V	**I**	**E**	**T**	A	**I**	**R**	**N**	**L**	**A**	G	**E**	A	A	N	**F**	S	N	D	**Y**	**A**	**V**	**I**	K	Y	R	I	C
V1/S	C	V	**I**	**E**	**T**	A	**I**	**R**	**N**	**L**	**F**	G	K	A	**T**	**Y**	Y	S	N	D	**Y**	**A**	**V**	**I**	K	Y	R	I	C
W2	C	V	**I**	**E**	**T**	A	**I**	**R**	**N**	I	**F**	G	K	A	**S**	**Y**	Y	S	N	D	**Y**	**A**	**V**	**I**	K	Y	R	I	C
7G8	**S**	V	M	N	**T**	A	**I**	C	**N**	I	S	G	K	A	A	N	**F**	**C**	**D**	**Y**	H	S	I	F	K	Y	R	I	C
Clinical isolates																													
RKL9610	C	V	M	N	K	A	N	**R**	**N**	I	S	**A**	K	A	A	N	**F**	S	N	D	H	S	I	F	K	Y	R	I	C
RKL10551	C	V	**I**	**E**	**T**	A	N	**R**	**N**	I	S	**A**	K	A	A	N	Y	S	N	D	**Y**	**A**	**V**	F	K	Y	R	I	C
RKL10868	C	V	M	N	K	A	N	C	S	I	S	**A**	K	A	A	N	Y	S	N	D	H	S	I	F	K	Y	R	I	C
RKL12330^*b*^	C	V	**I**	**E**	**T**	A	N	**C/R**	**S/N**	I	S	**A**	K	A	A	N	**Y/F**	S	N	D	**Y**	**A**	**V**	F	K	Y	R	I	C
RKL50072	C	V	M	N	K	A	N	**R**	**N**	I	S	**A**	K	A	A	N	**F**	S	N	D	H	S	I	F	K	Y	R	I	C
RKL50617	C	V	**I**	**E**	**T**	A	N	C	S	I	S	**A**	K	A	A	N	Y	S	N	D	H	S	I	F	K	Y	R	I	C
RKL58812^*b*^	C	V	M	N	K	A	N	**C/R**	**S/N**	I	S	**A**	K	A	A	N	**F**	S	N	D	**H/Y**	**S/A**	I	F	K	Y	R	I	C
RKL58925	C	V	M	N	K	A	N	**C/R**	**S/N**	I	S	**A**	K	A	A	**Y**	Y	S	N	D	**Y**	**A**	**V**	F	K	Y	R	I	C
RKL59175	C	V	M	N	K	A	N	**C/R**	**S/N**	I	S	**A**	K	A	A	N	**Y/F**	S	N	D	H	S	I	F	K	Y	R	I	C
RKL59290	C	V	**I**	**E**	**T**	A	N	C	S	I	S	**A**	K	A	A	N	Y	S	N	D	**Y**	**A**	**V**	F	K	Y	R	I	C
RKL59796	C	V	**I**	**E**	**T**	A	N	**R**	**N**	I	S	**A**	K	A	A	N	Y	S	N	D	H	S	I	F	K	Y	R	I	C
RKL60303	C	V	**I**	**E**	**T**	A	N	C	S	I	S	**A**	K	A	A	**Y**	Y	S	N	D	**Y**	**A**	**V**	F	K	Y	R	I	C
RKL60308	C	V	M	N	K	A	N	C	S	I	S	**A**	K	A	A	**Y**	Y	S	N	D	**Y**	**A**	**V**	F	K	Y	R	I	C
RKL60355^*b*^	C	V	**I**	**E**	**T**	A	N	**C/R**	**S/N**	I	S	**A**	K	A	A	N	**Y/F**	S	N	D	**H/Y**	**S/A**	**I/V**	F	K	Y	R	I	C
RKL61495	C	V	M	N	K	A	N	**R**	**N**	I	S	**A**	K	A	A	N	**F**	S	N	D	**Y**	**A**	**V**	F	K	Y	R	I	C
RKL64249	C	V	M	N	K	A	N	C	S	I	S	**A**	K	A	A	N	Y	S	N	D	**Y**	**A**	**V**	F	K	Y	R	I	C

a The P. falciparum 3D7 genome was used as a reference, and predicted amino acid substitutions at key positions implicated in drug resistance are highlighted in bold.

b Samples that are proposed to be multiclonal infections based on the frequency of heterozygous variants.

### ACT.

Sequence polymorphisms in the *Pfk13*, *Pfmdr1*, and *Pfmrp1* genes have been found to play a role in ACT resistance. Artemisinin combination therapy (ACT) comprises a combination of artemisinin-derived compounds, such as artesunate or artemether, with other longer-acting antimalarials to limit selection of parasites resistant to either drug (37). It was discovered recently that mutations in the propeller domain of the kelch protein PfK13 are associated with resistance to artemisinin derivatives in field isolates (38), and they are also able to confer *in vitro* resistance to artemisinin, albeit in specific genetic backgrounds (39). Amino acid substitutions in PfK13, including C580Y, Y493H, and R539T, among others (40), have been shown to correlate with slow clearance of parasites in patients treated with ACT in field isolates from Cambodia (38). Another *Pfk13* mutation, M476I, was identified in laboratory strains that acquired artemisinin resistance when subjected to drug pressure (38). A past survey of 384 samples across India identified four nonsynonymous SNPs in the propeller regions of the *Pfk13* gene, namely, G533A, S549Y, R561H, and A578S (41). We did not identify these or any other nonsynonymous SNPs in the K13 propeller domain of 16 P. falciparum clinical isolates from Raurkela. The K189T substitution, which lies outside the propeller domain, was detected in three MR4 strains (HB3, GB4, and D6) and three clinical isolates (RKL58812, RKL61495, and RKL50617). This substitution is widely prevalent in African artemisinin-sensitive isolates (42–44) and has also been seen in South Asian isolates (45). Only one synonymous SNP, L119L, was found in the isolate RKL60303. Based on their genotype, all the clinical isolates in our study are sensitive to artemisinin.

Artesunate (AS) is partnered with sulfadoxine-pyrimethamine (SP), called AS-SP, in most regions in India, except the northeastern region, where artemether is paired with lumefantrine (AL) due to widespread resistance against SP (31). The wild-type alleles of *Pfmdr1* coding for the amino acids N86 and D1246 are associated with resistance to AL (46, 47). The N^86^F^184^D^1246^ haplotype associated with AL treatment failure (48) was observed in seven clinical isolates and two reference strains, HB3 and TM91C235. The triple-mutant *Pfmdr1* allele encoding the substitutions C^1034^D^1042^Y^1246^, which confer increased sensitivity to artemisinin *in vitro* (49), was absent in all samples, except the 7G8 reference strain. In the case of *Pfmrp1*, all 16 clinical isolates and seven of the reference strains showed the F1390 allelic variant that is associated with decreased *in vitro* susceptibility to artemisinin, mefloquine, and lumefantrine (50). The amino acid substitution I876V in *Pfmrp1*, which was previously reported in recrudescent infections in East Africa following AL treatment (51), was present in nine clinical isolates.

### SP.

Sequence polymorphisms in the *Pfdhfr*, *Pfdhps*, and *Pfmrp1* genes have been found to play a role in SP resistance. Resistance to pyrimethamine (PYR) is most strongly associated with the S108N substitution in *Pfdhfr* (52, 53), which reduces the binding of PYR to the dihydrofolate reductase (DHFR) protein. Additional amino acid substitutions at C59R, N51I, and I164L further increase resistance to PYR (54). The triple mutant A^16^I^51^R^59^N^108^I^164^ and quadruple mutant A^16^I^51^R^59^N^108^L^164^ confer the highest level of resistance to PYR. These were detected in the PYR-resistant reference strains Dd2, TM91C235, V1/S, and W2. Five clinical isolates from Raurkela contain double mutants resulting in the A^16^N^51^R^59^N^108^I^164^ haplotype, which is similar to the PYR-resistant K1 strain. Six isolates possess the wild-type A^16^N^51^C^59^S^108^I^164^ haplotype, as seen in the PYR-sensitive NF54 strain. The remaining five isolates have two allelic variants at the *Pfdhfr* locus, as depicted in Table 2. Five key substitutions in *Pfdhps*, A437G, S436A, K540E, A581G, and A613T (55, 56), have been proposed to reduce the binding of the PfDHPS protein to its inhibitor sulfadoxine (SUL). The reference strain 3D7 has a single substitution, A437G, conferring moderate resistance to SUL (57). All 16 clinical isolates possess the SUL-sensitive S^436^A^437^K^540^A^581^A^613^ haplotype, observed in the reference strains D10, HB3, and FCB. The *Pfdhfr* and *Pfdhps* genotypes together indicate that all the clinical isolates are likely to be sensitive to SP chemotherapy. None of the clinical isolates contain the K1466R substitution in *Pfmrp1*, which has been proposed to increase sensitivity to SP by promoting the efflux of antifolates from the cell (58).

### CQ.

Sequence polymorphisms in the *Pfcrt* and *Pfmdr1* genes have been found to play a role in CQ resistance. Resistance to CQ is a result of key mutations in *Pfcrt* and *Pfmdr1* that reduce the accumulation of CQ in the parasite digestive vacuole (59, 60). The K76T substitution in *Pfcrt* is strongly associated with *in vitro* and *in vivo* resistance to CQ and is usually accompanied by additional mutations at amino acid positions 72 to 75, 97, 220, 271, 326, 356, and 371 (61–63). The K76T substitution was observed in seven of the clinical isolates as part of the C^72^V^73^I^74^E^75^T^76^ haplotype that is also present in a majority of the CQ-resistant reference strains. Nine clinical isolates had the C^72^V^73^M^74^N^75^K^76^ haplotype present in the CQ-sensitive strains NF54 E, D6, D10, and HB3. Interestingly, the most prevalent haplotype in India is the CQ-resistant S^72^V^73^M^74^N^75^T^76^ haplotype (64). However, its frequency varies from the western regions of the country (where it is fixed in the population) to the eastern regions, which are adjacent to Southeast Asian countries, such as Myanmar (65, 66). The S^72^V^73^M^74^N^75^T^76^ haplotype was not detected in any of the samples except the 7G8 reference strain from Brazil. Mutations causing substitutions in *Pfmdr1* codons 86, 184, 1034, 1042, and 1246 are linked with increased resistance to CQ (59, 67). The CQ-sensitive *Pfmdr1* haplotype N^86^Y^184^S^1034^N^1042^D^1246^ was detected in six of the clinical isolates and three reference strains, NF54 E, D6, and D10. Four clinical isolates possessed a single substitution, Y184F, similar to that observed in TM91C235, while three clinical isolates had two allelic variants at the Y184 position. Three clinical isolates had the CQ resistance-conferring N86Y substitution, similar to the K1, V1/S, and W2 reference strains. The H191Y substitution in PfMRP1, associated with increased susceptibility to CQ (68), was seen in eight of the 16 clinical isolates and two isolates that also had the wild-type H191 allelic variant.

### Using heterozygous variants for identification of mixed genotypes within samples.

**FIG 3:**
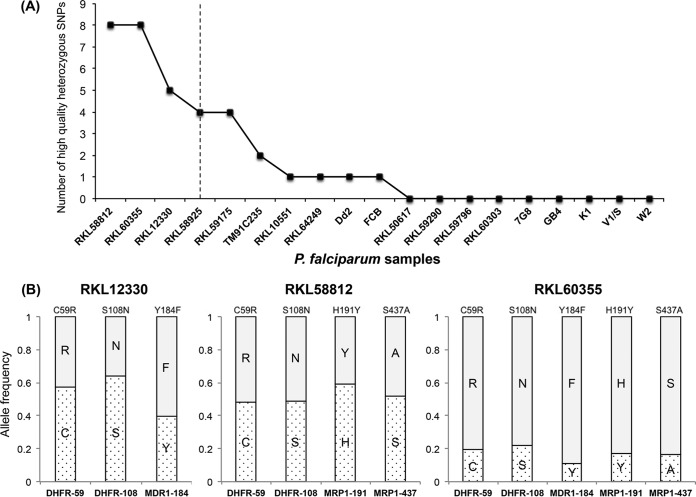
Estimation of mixed genotypes using heterozygous variant calls. (A) Plot showing number of high-quality heterozygous variant calls (*y* axis) for each P. falciparum sample (*x* axis). Isolates on the left of the vertical dotted line, which represents twice the median number of heterozygous variants observed in the entire data set, were classified as potential multiclonal infections. (B) Box plots representing the proportion of the two allelic variants (shaded or dotted) observed in heterozygous SNPs associated with drug resistance in samples RKL12330, RKL58812, and RKL60355, which were classified as multiclonal infections in the plot in panel A.

The depth of sequence coverage generated by the Ion Torrent PGM should theoretically enable the identification of mixed-genotype populations within samples. Indeed, during data analysis, we detected two variants, N86Y (36%) and N86F (64%), at *Pfmdr1* codon 86 in our Dd2 sample obtained from MR4. The N86F variant has been reported in field isolates from Africa and Afghanistan (69, 70), as well as the South Asian reference isolate Dd2 (11, 71). It appears that the two allelic variants observed in our Dd2 sample represent two copies of *Pfmdr1*, which have been observed in previous studies (11). Interestingly, the copy number variation of *Pfmdr1* has been implicated in reduced sensitivity to antimalarial drugs, such as CQ, mefloquine, and lumefantrine (72–74). This finding encouraged us to use heterozygous variant calls for the identification of multiclonal infections in the clinical isolates. Samples were ranked based on the total number of high-quality heterozygous SNPs (Fig. 3A). As might be expected due to a loss of diversity during *in vitro* culture adaptation, heterozygous SNPs were not detected in most of the P. falciparum reference strains. Four of the clinical isolates also did not exhibit any heterozygous variants, indicating the presence of a single parasitic clone in these isolates. On the other hand, the number of heterozygous SNPs in three of the samples, RKL12330, RKL58812, and RKL60355, was more than twice the median value observed in the entire data set (Fig. 3A). Further analysis of key drug resistance mutations with heterozygous variant calls revealed that the proportion of the reference and alternate allele was consistent across the various SNPs for three samples (Fig. 3B). Therefore, we classified three of the clinical isolates as multiclonal infections, comprising a mixture of genotypes with various drug susceptibilities. These results demonstrate the potential of the amplicon sequencing method to estimate the complexity of infection in P. falciparum clinical isolates. Although we only used high-quality heterozygous variants (allele frequency, >10%) for the purpose of this analysis, we should be able to improve the limit of detection of our NGS approach to identify rare variants (<1%) while accounting for sequencing errors and other technical artifacts.

## DISCUSSION

Malaria continues to be challenging to eradicate, in part because P. falciparum parasites have developed resistance to nearly every antimalarial drug developed (75, 76). After its introduction in 1934, chloroquine (CQ) was successfully used to treat malaria cases worldwide until the 1950s, when CQ-resistant P. falciparum arose in Southeast Asia and South America and eventually spread to India in the 1970s. Although its use against P. falciparum was discontinued, CQ remained the first-line treatment for P. vivax malaria in India (31). P. falciparum also developed resistance to subsequent antimalarial treatments involving combinations of antifolates, sulfadoxine, and pyrimethamine. Artemisinin combination therapy (ACT) was introduced in India in 2005, and artemisinin (ART) derivatives proved to be the most effective drugs developed against P. falciparum. However, in 2008, ART resistance was identified for the first time in Cambodia (77, 78) and has spread and arisen independently in other parts of Southeast Asia since then (79). The recent identification of mutations in the kelch propeller domain of the *Pfk13* gene as key causal determinants of artemisinin resistance have provided the opportunity for real-time surveillance of the emergence and spread of ART resistance in regions that are endemic for malaria. The detection of multiple *Pfk13* mutations associated with delayed parasite clearance in Myanmar (80), which shares a border with Northeast India, has raised the alarm for the spread of ART resistance to India, similar to the suspected trajectory of CQ- and SP-resistant parasites. So far, *Pfk13* surveillance studies in India have not yet detected any evidence for ART resistance in the country (41, 81). The success of ACT relies on the continued efficacy of ART and its partner drugs and is crucial for malaria control in India.

In order to address the pressing need to strengthen malaria surveillance, we have proposed here a next-generation amplicon sequencing method to enable high-throughput detection of genetic mutations in six P. falciparum genes associated with antimalarial drug resistance in India. The method can be easily adapted to include other candidate genes depending on the malaria treatment regimen used across different regions that are endemic for the disease. While most traditional surveillance methods focus on the detection of a limited number of known polymorphisms, our method involves sequencing the entire gene, which facilitates the discovery of novel polymorphisms involved in drug resistance. For example, the novel mutation (C350R) in *Pfcrt*, causing a reversal of CQ resistance conferred by the SVMNT haplotype in P. falciparum parasites from French Guiana, would have not been identified if only a limited number of SNPs had been analyzed (82). Moreover, as multiple genes can be associated with resistance to a single drug, the simultaneous analysis of these genes will improve our knowledge of the possible combinatorial effects of multiple mutations on parasite drug resistance. By monitoring the molecular markers of resistance for all drugs administered in a particular region, changes to treatment policy can be implemented to contain the spread of drug resistance. This is particularly relevant in the context of reintroducing old drugs in areas where previously resistant parasites have reverted to carrying drug-susceptible alleles in the prolonged absence of drug pressure, as recently observed in Malawi with the return of the CQ-sensitive wild-type *Pfcrt* allele in the population a decade after CQ withdrawal (83). We also demonstrated the potential of amplicon sequencing to discriminate between single and multiclonal infections. As malaria parasite infections can consist of more than one parasite clone, the presence of both drug-sensitive and drug-resistant parasites within a single patient may occur. We plan to study the variation in within-host diversity in response to chemotherapy by obtaining patient samples before and after drug treatment, so as to track changes in the frequency of individual alleles identified in multiclonal infections. Analysis of the amplicon sequencing results in conjunction with clinical and epidemiological data associated with the patient isolates can help us decipher the impact of the complexity of infection on drug susceptibility or disease phenotype.

As proof of principle, we applied the method to 28 P. falciparum samples at a cost of $95 per patient, but the technology can allow multiplexing of up to 96 samples in a single run, significantly reducing the cost to approximately $73 per patient sample ($14 for DNA extraction and generation of the amplicon pool, and $59 for library preparation and sequencing). The use of the Ion Torrent platform makes our technique particularly suitable in countries endemic for malaria that have limited computational infrastructure and lack of advanced training in bioinformatics, as the instrument comes equipped with a suite of data analysis tools for read alignment, coverage, and variant detection that can be operated with minimum information technology expertise. As patient blood samples can be collected, transported, and stored for a long time without the need for *in vitro* culture, national malaria control programs could implement large-scale amplicon sequencing studies that are not feasible using *in vivo* and *in vitro* assays. As part of the National Institutes of Health-funded International Centers of Excellence for Malaria Research initiative, we have set up the Ion Torrent PGM platform at the National Institute for Malaria Research in Delhi for in-house sequencing of Plasmodium samples (24). We are currently using this method for multiplexed sequencing of clinical isolates collected from epidemiologically diverse sites in India (84).

## Supplementary Material

Supplemental material
