# Supplementary material for: A Method for Amplicon Deep Sequencing of Drug Resistance Genes in Plasmodium falciparum Clinical Isolates from India
Source: J Clin Microbiol. 2016 May 23;54(6):1500–11. doi: 10.1128/JCM.00235-16 (PMC4879288; doi:10.1128/JCM.00235-16)
Supplement: Supplemental material [file supp_54_6_1500__index.html]

A Method for Amplicon Deep Sequencing of Drug Resistance Genes in Plasmodium falciparum Clinical Isolates from India — Supplemental material 

# A Method for Amplicon Deep Sequencing of Drug Resistance Genes in Plasmodium falciparum Clinical Isolates from India

## Supplemental material

- Supplemental file 1 -

  Fig. S1 (Schematic representation of amplicons from six drug resistance genes)

  PDF, 534K
- Supplemental file 2 -

  Tables S1 (Primer sequences and genome coordinates of six *P. falciparum* target genes), S2 (Overview of multiplexed sequencing output, read alignment, and coverage statistics), S3 (List of SNPs reported by the Ion Torrent PGM Variant Caller plugin), S4 (List of high-quality coding SNPs in six *P. falciparum* target genes for 28 samples), and S5 (Summary of additional SNPs in six *P. falciparum* target genes)

  XLSX, 133K
