# Supplementary material for: A Method for Amplicon Deep Sequencing of Drug Resistance Genes in Plasmodium falciparum Clinical Isolates from India
Source: J Clin Microbiol. 2016 May 23;54(6):1500–11. doi: 10.1128/JCM.00235-16 (PMC4879288; doi:10.1128/JCM.00235-16)
Supplement: Supplemental material [file JCM.00235-16_zjm999094982so1.pdf]

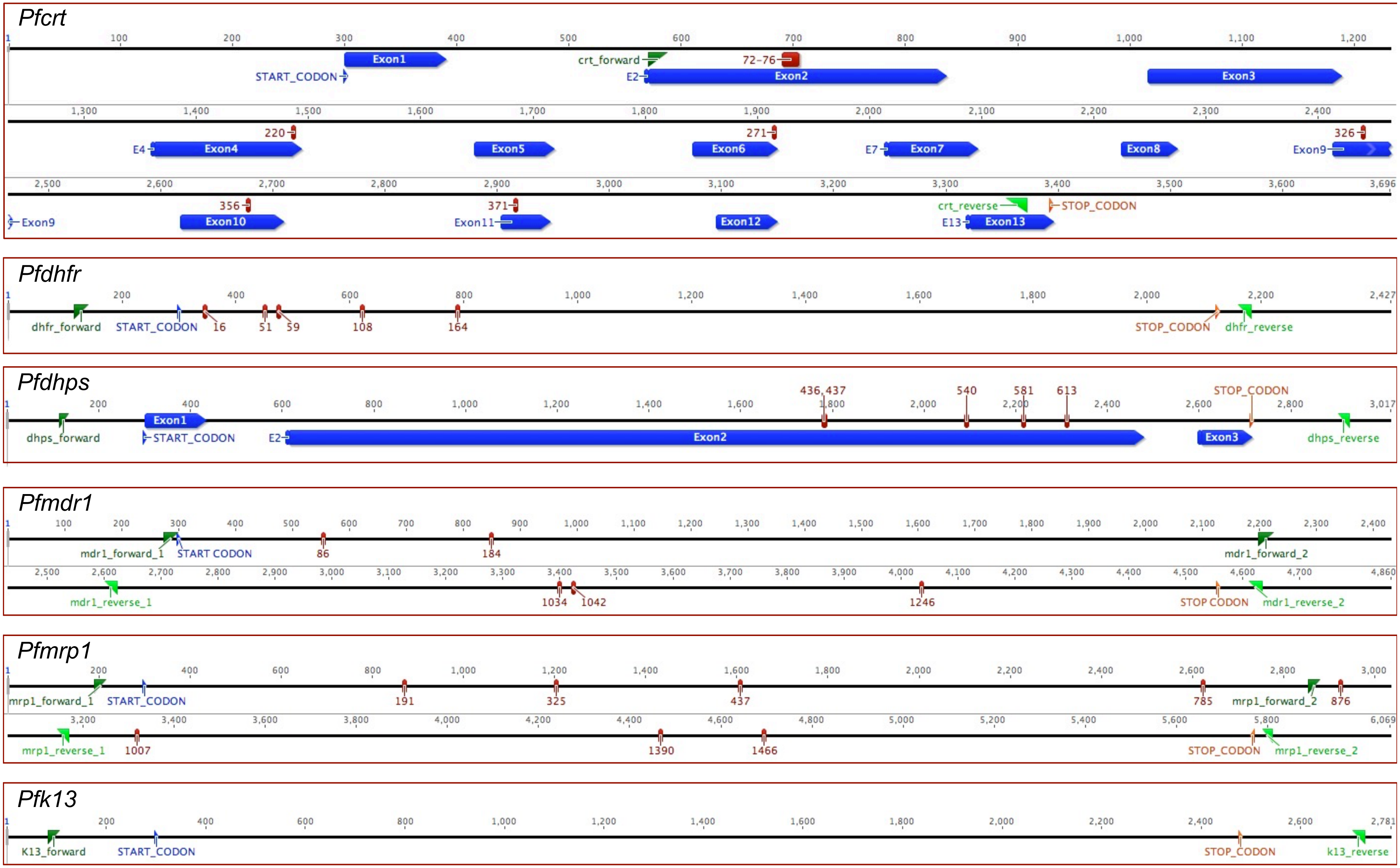

Figure S1. Schematic representation of amplicons from six drug resistance genes.
The diagrams shown for each gene also include 300 bp flanking sequencing around the CDS. Amplicon primers are annotated in dark green (forward primers), and light green (reverse primers). Exons are highlighted in blue, and coordinates of the start and stop codons have been annotated in blue and brown, respectively. Positions marked in red correspond to codons containing mutations known to be associated with drug resistance.
